# Thermally Conductive Film Fabricated Using Perforated Graphite Sheet and UV-Curable Pressure-Sensitive Adhesive

**DOI:** 10.3390/nano11010093

**Published:** 2021-01-03

**Authors:** Hee-Jin Lee, Gayoung Lim, Eunseong Yang, Young-Seok Kim, Min-Gi Kwak, Youngmin Kim

**Affiliations:** Display Research Center, Korea Electronics Technology Institute, 25 Saenariro, Bundang-gu, Seongnam 13509, Korea; gmlwls5010@gmail.com (H.-J.L.); addzero@kakao.com (G.L.); didguswjd567@naver.com (E.Y.); vis4freedom@keti.re.kr (Y.-S.K.); kwakmg@keti.re.kr (M.-G.K.)

**Keywords:** thermally conductive film, perforated graphite sheet, UV-curable adhesive, U-folding test

## Abstract

Thermally conductive films play a crucial role in expanding the lifetime of electronics by dissipating concentrated heat to heatsinks. In this work, a thermally conductive film (g-TC film) was manufactured using a perforated graphite sheet (p-GS) and a UV-curable pressure-sensitive adhesive (PSA) by lamination. A novel UV-curable PSA was prepared by incorporating a UV-curable abietic acid ester into a PSA composition. The UV-curable PSA became a tack-free film upon UV irradiation; thus, a flexible g-TC film with a 52-μm thickness was obtained. The defects in the g-TC film caused by air bubbles were removed by treating the p-GS with oxygen plasma. As the UV-cured PSA made a joint through the holes in the p-GS, cleavage of the graphite was not observed after 10,000 U-folding test cycles with a folding radius of 1 mm. The calculated in-plane thermal conductivity of the fabricated g-TC film was 179 W∙m^−1^K^−1^, which was stable after the U-folding tests.

## 1. Introduction

The accumulation of heat generated at heat sources causes malfunctions of electronics, which are severe for portable devices with small and densely packed chips [1]. To address this problem, materials with high thermal conductivity have been employed for thermal management. Because heat flows from the heat source to the heat sink through thermally conductive (TC) materials, the higher thermal conductivity TC materials have, the more efficiently the heat is dissipated [2]. In particular, carbon-based materials [3], such as carbon nanotubes (CNTs), graphene [4,5,6,7,8], and graphite [1,4,9], have been studied for thermal management due to their excellent thermal conductivity. However, polymer/carbon filler composites show rather low thermal conductivity because of the thermal resistance at the polymer/filler interfaces. To minimize phonon scattering at the interfaces, a free-standing graphene film [10,11] was fabricated and embedded in a polymer matrix [5]. The thermal conductivity of the composite was increased by two orders of magnitude compared to that of polymer because the 2D structure of graphene connected by sp^2^ hybridized carbon is advantageous for phonon transfer. Despite this advantage, the use of graphene film is limited because of the troublesome process of exfoliation of graphite into graphene [5]. On the contrary, graphite sheets (GSs) manufactured by thermal decomposition of a polyimide film [12,13,14] are commercially available. Because graphite sheets spread heat efficiently, they are commonly used as TC materials for electronics, such as smartphones, tablets, and televisions. The need for pliable TC films is increasing as displays become lighter, compact, and flexible. In this work, we developed a method to fabricate a flexible TC film using a UV-curable pressure-sensitive adhesive (PSA) by lamination. To prevent separation of the graphite layer, a perforated graphite sheet (p-GS) was employed. Due to its viscoelastic properties, the PSA wets the surface of the graphite sheet instantly upon slight pressure and flows to fill the holes in it, which increases the mechanical stability of the graphite. Because lamination does not require any external energy, it is widely applied for producing tapes in the industrial field. Unlike conventional PSAs, the UV-curable PSAs become non-sticky upon UV irradiation owing to the crosslinking of the multifunctional acrylates [15,16,17]. We designed and synthesized a novel UV-curable abietic acid ester and used it for fabricating a UV-curable PSA tape. The addition of the abietic acid esters not only increases the initial peeling adhesion of the UV-curable PSA, but also leads to dramatic reduction in peel force after UV treatment. This high initial peel adhesion is beneficial to achieve a strong bond between the graphite sheet and PSA layer. A p-GS embedded film (g-TC) was fabricated by irradiating the sandwich structure of PSA-U3/p-GS/PSA-U3 with a UV light. This process was further optimized by employing O_2_ plasma treatment, which realized an air-bubble-free film. The effect of perforation on the cleavage of the graphite was investigated through a tape test. The change in the thermal conductivity of the g-TC film after a U-bending test was evaluated as well.

## 2. Materials and Methods

### 2.1. Materials

Butyl acrylate (>99%), 2-ethylhexyl acrylate (>99%), ethyl acrylate (>99%), 2-hydroxyethyl acrylate (>95%), acrylic acid (>99%), abietic acid (>80%), triethylamine (>99%), and 2-isocyanatoethyl methacrylate (>98%) were purchased from Tokyo Chemical Industry. Tris(4-hydroxyphenyl)methane triglycidyl ether (>95%) and dibutyltin dilaurate (95%) were purchased from Sigma-Aldrich Korea Ltd. (Yongin, Korea). Toluene (99.5%), ethyl acetate (99.5%), and methyl ethyl ketone (99.5%) were purchased from Samchun Chemical (Pyeongtaek, Korea). Azobisisobutyronitrile (AIBN, 98%) was purchased from Junsei Chemical (Tokyo, Japan). All chemicals were used as received without further purification. The epoxy crosslinker was used after dilution to a solid content of 5 wt% with toluene. A 17-μm-thick graphite sheet was purchased from Tanyuan technology (Changzhou, China). A silicone-coated polyethylene terephthalate (PET) film and a polyethylene terephthalate (PET) film were purchased from SKC (Seoul, Korea). A stainless steel (SS) plate was purchased from MMSTECH (Bucheon, Korea). The micro structure of the g-TC film was analyzed using a scanning electron microscope (SEM, JSM 6380, JEOL, Tokyo, Japan) and a digital microscope (MXB-5000REZ, HIROX, Tokyo, Japan).

### 2.2. Instrumentation

The ^1^H and ^13^C NMR spectra were measured on a nuclear magnetic resonance (NMR) spectrometer equipped with Bruker Top Spin 3.2 software (AscendTM 400, Bruker, Madison, WI, USA). The Fourier transform infrared (FTIR) spectra in the range from 600 to 4000 cm^−1^ were obtained by the attenuated total reflectance method using an FTIR spectrophotometer (IRAffinity-1S, Shimadzu, Kyoto, Japan). The glass transition temperature (Tg) and molecular weights were measured using a DSC-4000 (PerkinElmer, Waltham, USA) and Agilent 1100 (Agilent, Santa Clara, USA), respectively. The peel strength was measured on a tensile tester (HZ-1007E, MMSTECH, Bucheon, Korea). The viscosity was measured on a Sine-wave Vibro Viscometer (A&D Company, Tokyo, Japan). The frequency dependence of the shear storage modulus (G′) and shear loss modulus (G″) was measured using a rheometer (MCR102, Anton Parr, Graz, Austria). The temperature dependence of the extension storage modulus (E′) was measured using a dynamic mechanical analyzer (DMA8000, Perkin Elmer, Waltham, USA). The thermal diffusivity and specific heat capacity were measured using a LFA 447 nanoflash (NETZSCH, Selb, Germany) and a DSC 214 Polyma (NETZSCH, Selb, Germany).

### 2.3. Synthesis of Acrylic Polymer 1 (AP-1)

A flask was charged with butyl acrylate (75 g), 2-ethylhexyl acrylate (45 g), ethyl acrylate (15 g), 2-hydroxyethyl acrylate (1.5 g), acrylic acid (13.5 g), AIBN (0.45 g), ethyl acetate (150 g). The mixture was heated at 80 °C in an oil bath. When the solution was boiled vigorously, the flask was taken out from the oil bath and kept at room temperature. Once the boiling subsided, the solution was refluxed at 80 °C for 8 h. After the solution was cooled to room temperature, toluene (37.5 g) was added, which afforded a viscous solution containing acrylic polymer 1 with a solid content of 45 wt%. The final solution was designated as solution-A.

### 2.4. Synthesis of Abietic Acid Ester 2 (AAE-2)

A flask was charged with abietic acid (3.94 g, 13.0 mmol), tris(4-hydroxyphenyl)methane triglycidyl ether (2.0 g, 4.3 mmol), and triethylamine (0.06 g, 0.6 mmol). To this mixture, toluene (5.94 g) was added to afford a yellow solution. After it was heated at 100 °C for 12 h, it was cooled to room temperature to produce a solution containing abietic acid ester 2 with a solid content of 50 wt%. This solution was designated as solution-B.

^1^H NMR (400 MHz, CDCl_3_) data: δ: 7.00-6.82 (m, aromatic ring), 5.77 (s, HC=C), 5.34 (bs, H-C=C), 4.27-4.22 (m), 4.00-3.80 (m), 2.24-0.83 (aliphatic chain).

^13^C NMR (100 MHz, CDCl_3_) data: δ: 178.8 (C(O)O), 156.7, 145.3 (C=C), 135.6, 135.5 (C=C), 130.3, 130.1, 122.4 (C=C), 120.4 (C=C), 114.5, 114.3, 68.8, 65.5, 50.9, 46.7, 45.1, 38.3, 37.2, 34.9, 34.5, 27.4, 25.7, 22.5, 20.9, 18.1, 17.1, 14.1.

### 2.5. Synthesis of Tri-Methacrylate 3 (TM-3)

The flask was charged with abietic acid ester 2 (1.0 g, 0.8 mmol), 2-isocyanatoethyl methacrylate (0.27 g, 1.74 mmol), methyl ethyl ketone (1.27 g), and dibutyltin dilaurate (cat.). The mixture was heated at 60 °C for 3 h and then cooled to room temperature. After all volatile materials were removed from the solution, the brown powder was washed with hexanes twice followed by drying at 50 °C overnight to afford tri-methacrylate (TM-3).

^1^H NMR (400 MHz, CDCl_3_) data: δ: 6.93-6.76 (m, aromatic ring), 6.09 (m, H_2_C=C), 5.70 (bs, HC=C), 5.56 (m, H_2_C=C), 5.34-5.25 (bs, HC=C), 4.33-3.84 (m), 4.20 (t, OCH_2_CH_2_N), 3.47 (m, OCH_2_CH_2_N), 2.20-0.78 (aliphatic chain).

^13^C NMR (100 MHz, CDCl_3_) data: δ: 178.0 (C(O)O), 167.5, 167.2, 158.0, 156.7, 155.5, 145.3, 136.0, 135.9, 135.6, 130.2, 126.1, 126.0, 122.4, 120.4, 114.3, 64.1, 50.9, 46.7, 45.2, 40.2, 39.6, 38.3, 37.0, 36.9, 34.5, 34.4, 27.4, 25.6, 22.5, 20.9, 18.3, 18.1, 17.0, 14.0.

### 2.6. Fabrication of PSA-T2 Tape

A mixture of solution-A (4.0 g), an epoxy crosslinker (36 mg), and solution-B (0.54 g) was bar-coated on a silicone-coated PET film (50 μm) using an applicator. The coating layer was dried at 120 °C for 90 s to produce a 20-μm-thick adhesive layer on the silicone-coated PET film. The adhesive film was laminated with a PET film (50 μm) and kept at 80 °C for 1 day to afford the PSA-T2 tape.

### 2.7. Fabrication of PSA-U3 Tape

A mixture of solution-A (2.0 g), an epoxy crosslinker (18 mg), TM-3 (0.45 g), and irgacure-184 (54 mg) was bar-coated on a silicone-coated PET film (50 μm) using an applicator. The coating layer was dried at 120 °C for 90 s to fabricate a 20-μm-thick adhesive layer on a silicone-coated PET film. The adhesive film was laminated with a PET film (50 μm) using a 2-Kg rubber roller and kept at room temperature for 1 day to produce the PSA-U3 tape.

### 2.8. Fabrication of g-TC Film

**Figure 1:**
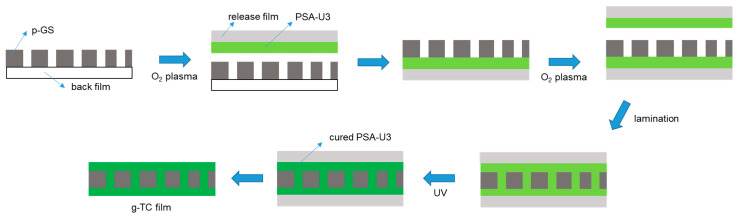
Schematic process for fabrication of a g-TC film.

The process for fabricating a g-TC film, in which a perforated graphite sheet (p-GS) film was embedded, is depicted in Figure 1. A mixture of solution-A (2.0 g), an epoxy crosslinker (18 mg), TM-3 (0.45 g), and irgacure-184 (54 mg) was combined in a vortex mixer for 5 min. It was bar-coated on a silicone-coated PET film (50 μm) using an applicator and dried at 120 °C for 90 s to afford a PSA-U3/release film. The p-GS on a back film was treated with oxygen plasma for 300 s, laminated to the PSA-U3/release film, then the back film was removed to afford the structure of p-GS/PSA-U3/release film. The graphite side of this film was treated with oxygen plasma and laminated into another PSA-U3/release film to produce the structure of release film/PSA-U3/p-GS/PSA-U3/release film. The removal of the release films from both the sides of the multilayer film generated the g-TC film in which p-GS was embedded.

### 2.9. Peel Force Test

As-prepared PSA-T2 and PSA-U3 tapes were cut into strips 1 in width. After removal of the release film, the tapes were laminated to an SS plate and kept at room temperature for 30 min. The peel forces of the tapes were measured by a 180° peel test at the peeling rate of 300 mm/min.

### 2.10. Thermal Diffusivity and Specific Heat Capacity Measurement

The thermal diffusivity was measured by laser flash analysis. For this, as prepared g-TC film was cut into a circle with a diameter of 1 in then the sample was placed in a stage and covered with a holder. The bottom face of the sample was irradiated using a Xenon lamp with a maximum pulse energy of 10 J, and the temperature of the top face of it was measured using a InSb infrared detector. The relative standard deviation of the measured thermal diffusivity was 1%. The specific heat capacity was measured according to the ASTM E-1269 standard method using a sapphire as a standard.

## 3. Results and Discussion

**Scheme 1:**
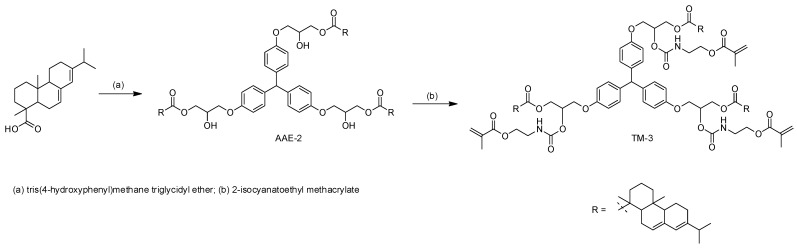
Synthesis of AAE-2 and TM-3.

Due to its low storage modulus (G’), a PSA wets a substrate intimately during bonding and is deformed during detachment, which leads to high peel adhesion [18,19]. The adhesive performance of PSAs have been further optimized using abietic acid esters, which act as plasticizers in PSAs [20,21]. In this work, we synthesized acrylic polymer (AP-1) and AAE-2 for the PSA compositions. AP-1 was prepared by copolymerizing acrylic monomers containing butyl acrylate and 2-ethylhexyl acrylate as the main components. The viscosity of the acrylic polymer solution was 4400 cps, and Tg of AP-1 was measured to be around −35 °C by differential scanning calorimetry. Number-average and weight-average molecular weights of AP-1 were determined to be 19 kg/mol and 260 kg/mol, respectively, by gel permeation chromatography. Next, abietic acid ester (AAE-2) was synthesized through the stoichiometric reaction between abietic acid and tris(4-hydroxyphenyl)methane triglycidyl ether (Scheme 1). The product was characterized by FTIR and NMR spectroscopy. The reduction in intensity at 910 cm^−1^ corresponding to the epoxide ring indicated that a ring-opening reaction had occurred (Figure S1) [22]. The strong IR absorption at 1720 cm^−1^ proved the presence of the ester bond in AAE-2. In the ^1^H NMR spectrum of AAE-2, the disappearance of proton resonances for glycidyl groups also demonstrated that ring-opening had occurred (Figure S2). The presence of the ester bond was confirmed by the peak at 178.8 ppm in the ^13^C NMR spectrum of AAE-2.

**Figure 2:**
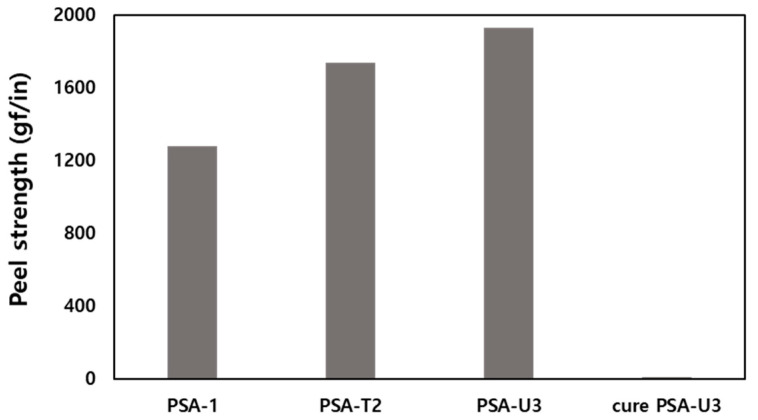
Peel strength of pressure-sensitive adhesive PSA-1, PSA-T2, PSA-U3, and the cure PSA-U3 tapes**.**

To investigate the effect of AAE-2 on the peel force, a PSA composition was prepared by mixing AP-1 and AAE-2 at the AP-1-to-AAE-2 weight ratio of 100 to 15. The composition was bar-coated on a release film and dried to afford a 20-μm-thick adhesive layer, which was transferred to a PET film to produce PSA-T2 tape. The measured peeling adhesion of the PSA-T2 tape was 1740 gf/in in a 180° peel test (Figure 2). Given that the peel strength of the PSA-1 tape without AAE-2 was 1280 gf/in, the addition of AAE-2 enhanced the peeling adhesion of PSA-T2 by 36%.

**Figure 3:**
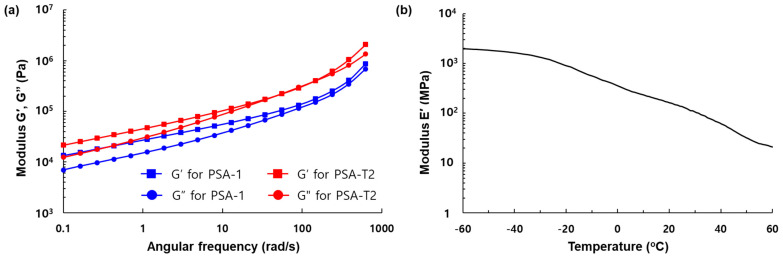
(**a**) G′, G″ against the angular frequency for PSA-1 and PSA-T2 measured by a rheometer; (**b**) E′ versus temperature of the cure PSA-U3.

The increased peel force of the PSA-T2 is explained by G′ and G″ against the angular frequency as shown in Figure 3a. To realize high peel force, the PSA should make good contact with the substrate during bonding and dissipate the applied energy by elastic deformation during detachment; thus, the ratio of G″ (ω_2_)/G′ (ω_1_), where ω_2_ and ω_1_ are the debonding and bonding frequencies, is proportional to the peel strength [23]. Angular frequencies of 386 rad/s and 1.13 rad/s were chosen for the debonding and bonding frequencies, respectively. The ratios of G″ (ω_2_)/G′ (ω_1_) for PSA-1 and PSA-T2 were calculated to be 12.3 and 17.2, respectively, which showed consistency with the results from the peel adhesion tests. Given that the G′s for both the PSAs were less than 100 kPa at the bonding frequency [24], the deformation of PSA-T2 mainly caused the increase in peel strength. The G″ at 386 rad/s for PSA-T2 was increased largely because AAE-2 serves as a plasticizer (G″ (348 kPa) for PSA-1 and G″ (813 kPa) for PSA-T2).

Next, the UV curable TM-3 containing abietic acid esters and methacrylates were newly synthesized. Although the addition of multifunctional (meth)acrylates to the PSA composition realizes peelable PSAs, many of them reduce the initial peel adhesion of the PSAs [17]; thus, the design and synthesis of novel UV curable compounds are crucial. The synthesized compound (TM-3) was characterized by FTIR and NMR spectroscopy. In the FTIR spectrum, IR absorption at 1635 and 817 cm^−1^ corresponding to H–C=C stretching proved the existence of methacrylates in TM-3 (Figure S3). The proton resonances at 5.56 and 6.09 ppm corresponding to H_2_C=C(Me)(CO) were observed in the ^1^H NMR spectrum of TM-3. The presence of the urethane bond in TM-3 was confirmed by the carbon resonance at 155.5 ppm in the ^13^C-NMR spectrum. To ascertain UV curing of methacrylates, TM-3 was mixed with a photo-initiator and subsequently irradiated with UV light. Unlike TM-3, the UV-cured sample was not soluble in organic solvents, indicating that polymerization had occurred. We fabricated a peelable PSA tape (PSA-U3) by admixing AP-1 with TM-3 at the AP-1-to-TM-3 weight ratio of 100 to 50. The peeling adhesion of the fabricated PSA-U3 tape was determined to be 1930 gf/in in a 180° peel test (Figure 2). Unlike commercially available acrylates [17], TM-3 did not degrade the adhesion properties of PSA-U3; rather, it increased the peel strength by 50% compared to PSA-1. Furthermore, the peel adhesion of PSA-U3 was dramatically reduced to 11 gf/in by 2 orders of magnitude upon UV irradiation. This can be attributed to the PSA-U3 hardening [25], which was ascertained by dynamic mechanical analysis. Because E′ of the cured PSA-U3 was 140 MPa at 25 °C (Figure 3b), which is much greater than Dahlquist criterion (<0.1 MPa) [26], the cured PSA-U3 did not show permanent tack; thus, the peel adhesion was dramatically decreased.

**Figure 4:**
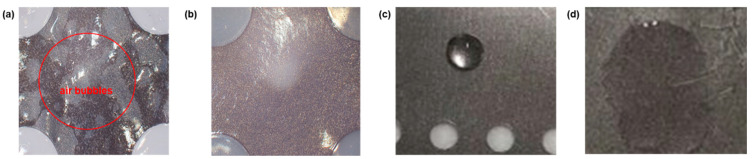
Photos of the surfaces of the g-TC films obtained (**a**) without and (**b**) with O_2_-plasma treatment; photos of water drops on the surfaces of the graphite sheets (**c**) before and (**d**) after oxygen plasma treatment**.**

A thermally conductive (g-TC) film in which a perforated graphite sheet (p-GS) was embedded was fabricated using PSA-U3 (Figure 1). Although commercially available graphite sheets have high thermal conductivity, they alone are not practically applicable because of graphite cleavage [27]. To address this problem, we manufactured the p-GS with 3-mm diameter holes by punching. The perforation accounts for about 15% of the p-GS area. Because the PSA-U3 fills the holes in the p-GS and makes a joint, the graphite is not readily cleaved. To manufacture the g-TC film, both sides of the p-GS were laminated to PSA-U3/release films followed by irradiation with UV light, after which the release films were removed. The hardening of PSA-U3 and removal of the release films made the g-TC film flexible. However, defects caused by air bubble trapping were observed in the g-TC film (Figure 4a). Given that the p-GS is hydrophobic (water contact angle was 91.6°, as shown in Figure 4c and Figure S4) [28], the adhesion of PSA-U3 to the graphite surface was low; thus, the hardening of PSA-U3 induced the detachment of the adhesive layer from the graphite sheet upon UV irradiation, leaving air bubbles at the interfaces. To resolve this problem, the p-GS was treated with oxygen plasma, which enhanced the adhesion of PSA-U3 to the graphite sheet (Figure 4b). After O_2_-plasma treatment, a water drop was completely absorbed by the graphite sheet, indicating that the hydrophobic p-GS had become hydrophilic (Figure 4d) [29]. This process ended up generating a defect-free g-TC film with a thickness of 52 μm, as shown in Figure S5. The in-plane thermal diffusivity of the g-TC film was determined to be 120 mm^2^/s by the laser flash method at 25 °C [30]. According to equation (1) [31], the in-plane thermal conductivity of the g-TC film was calculated to be 179 W∙m^−1^K^−1^ at 25 °C. Incorporation of the p-GS into the polymer enhances the thermal conductivity by 3 orders of magnitude compared to that (0.1–0.5 W∙m^−1^K^−1^) of polymers [32]. Given that the alignment of the graphene flakes by compression enhanced the thermal conductivity of the resulting film up to 90 W/mK in the previous study [10], the high thermal conductivity of the g-TC film was attributed to the highly ordered carbon atoms in the graphite sheet.

*k* = α∙*ρ*∙Cp (1)

where *k* is the thermal conductivity, α is the thermal diffusivity, *ρ* is the density, and Cp is the specific heat capacity.

**Figure 5:**
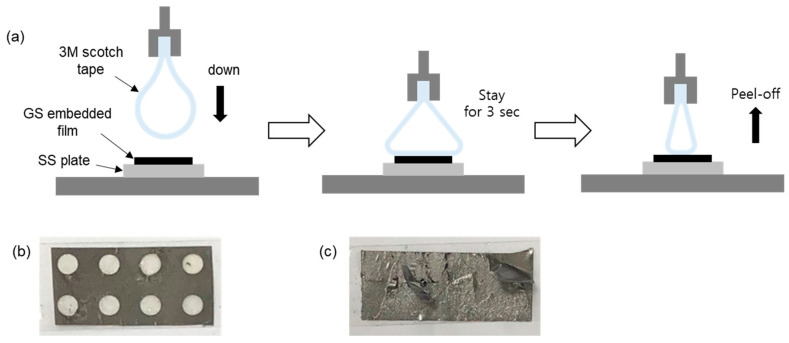
(**a**) Schematics for the tape testing method, and photos of the free-standing graphite film fabricated using (**b**) perforated and (**c**) non-perforated graphite sheet after the tape testing**.**

To investigate the effect of the perforation on the cleavage of the graphite, the g-TC was subject to a tape test (Figure 5a). For this purpose, 3M scotch tape with peeling adhesion of 180 gf/in was slowly (200 mm/min) applied to the g-TC film and was detached from it after 3 s of contact. No delamination of the graphite sheet was observed in the g-TC film after detachment (Figure 5b). On the contrary, when a graphite sheet without perforation was employed, the graphite was cleaved after the tape test (Figure 5c). This clearly indicates that the PSA-U3 filling the holes prevented the graphite sheet from being torn.

**Figure 6:**
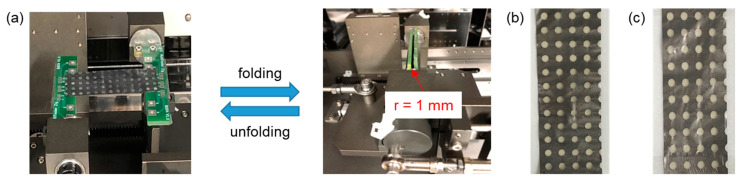
(**a**) Photos of the unfolded and folded g-TC films during the U-folding tests; photos of the g-TC films (**b**) before and (**c**) after 10,000 cycles of the U-folding tests**.**

The mechanical durability of the fabricated g-TC film was tested through a U-shape folding test (Figure 6). The film was cut into 18 mm × 58 mm samples, and both ends of the film were attached to two plates using double-sided adhesives. During the folding tests, the g-TC film was repeatedly folded and unfolded with a folding radius of 1 mm and a folding rate of 23 cycles/min. After 10,000 test cycles, no cracks or delamination were observed in the specimen with a scanning electron microscope (Figure S6), and the measured thermal conductivity was 170 W∙m^−1^K^−1^. This mechanical durability was attributed to the flexibility of the g-TC film.

## 4. Conclusions

In this work, a thermally conductive film (g-TC film), in which a perforated graphite sheet was embedded, was fabricated using a UV-curable PSA through a simple lamination process. Because the UV-curable PSA filled the holes in the p-GS, made a joint, and became a tack-free film, the g-TC film was flexible and mechanically stable. Trapped air bubbles stemming from the low surface energy of graphite were removed by treatment of the p-GS with O_2_-plasma. The thermal conductivity of the g-TC film was 179 W∙m^−1^K^−1^ and showed little change after 10,000 U-folding test cycles. This flexible and reliable g-TC film is expected to be applicable for solving the heat-related issues of flexible displays whose shapes are repeatedly changed. Last, but not least, the lamination process is suitable for the industrial production of thermally conductive films using previously installed equipment.

## Data Availability

Data sharing not applicable.

## Supplementary Materials

The following are available online at https://www.mdpi.com/2079-4991/11/1/93/s1, Figure S1: FTIR spectra of (a) tris(4-hydroxyphenyl)methane triglycidyl ether and (b) AAE-2, Figure S2: ^1^H-NMR spectra of (a) tris(4-hydroxyphenyl)methane triglycidyl ether and (b) AAE-2. The proton resonances for epoxide ring were disappeared through a ring-opening reaction, Figure S3: FTIR spectra of TM-3, Figure S4: A photography image of contact angle of a water drop on the graphite sheet. Figure S5: A cross sectional microscopic image of the g-TC film. Figure S6: SEM images of surfaces of the g-TC films (a) before and (b) after U-folding tests.

## References

[1] Chen W., Wu K., Liu Q., Lu M. (2020). Functionalization of graphite via Diels-Alder reaction to fabricate poly (vinyl alcohol) composite with enhanced thermal conductivity. Polymer.

[2] Kim J.-W., Lee D.H., Jeon H.-J., Jang S.I., Cho H.M., Kim Y. (2018). Recyclable thermosetting thermal pad using silicone-based polyurethane crosslinked by Diels-Alder adduct. Appl. Surf. Sci..

[3] Balandin A.A. (2011). Thermal properties of graphene and nanostructured carbon materials. Nat. Mater..

[4] Ohayon-Lavi A., Buzaglo M., Ligati S., Peretz-Damari S., Shachar G., Pinsk N., Riskin M., Schatzberg Y., Genish I., Regev O. (2020). Compression-enhanced thermal conductivity of carbon loaded polymer composites. Carbon.

[5] Bustero I., Gaztelumendi I., Obieta I., Mendizabal M.A., Zurutuza A., Ortega A., Alonso B. (2020). Free-standing graphene films embedded in epoxy resin with enhanced thermal properties. Adv. Compos. Hybrid Mater..

[6] Shahil K.M.F., Balandin A.A. (2012). Graphene–Multilayer Graphene Nanocomposites as Highly Efficient Thermal Interface Materials. Nano Letters.

[7] Barani Z., Mohammadzadeh A., Geremew A., Huang C.-Y., Coleman D., Mangolini L., Kargar F., Balandin A.A. (2020). Thermal Properties of the Binary-Filler Hybrid Composites with Graphene and Copper Nanoparticles. Adv. Funct. Mater..

[8] Kargar F., Barani Z., Salgado R., Debnath B., Lewis J.S., Aytan E., Lake R.K., Balandin A.A. (2018). Thermal Percolation Threshold and Thermal Properties of Composites with High Loading of Graphene and Boron Nitride Fillers. ACS Appl. Mater. Interfaces.

[9] Li G., Tian X., Xu X., Zhou C., Wu J., Li Q., Zhang L., Yang F., Li Y. (2017). Fabrication of robust and highly thermally conductive nanofibrillated cellulose/graphite nanoplatelets composite papers. Compos. Sci. Technol..

[10] Malekpour H., Chang K.H., Chen J.C., Lu C.Y., Nika D.L., Novoselov K.S., Balandin A.A. (2014). Thermal Conductivity of Graphene Laminate. Nano Letters.

[11] Renteria J.D., Ramirez S., Malekpour H., Alonso B., Centeno A., Zurutuza A., Cocemasov A.I., Nika D.L., Balandin A.A. (2015). Strongly Anisotropic Thermal Conductivity of Free-Standing Reduced Graphene Oxide Films Annealed at High Temperature. Adv. Funct. Mater..

[12] Inagaki M., Ohta N., Hishiyama Y. (2013). Aromatic polyimides as carbon precursors. Carbon.

[13] Takeichi T., Eguchi Y., Kaburagi Y., Hishiyama Y., Inagaki M. (1998). Carbonization and graphitization of Kapton-type polyimide films prepared from polyamide alkyl ester. Carbon.

[14] Venkatachalam S., Depriester M., Sahraoui A.H., Capoen B., Ammar M.R., Hourlier D. (2017). Thermal conductivity of Kapton-derived carbon. Carbon.

[15] Kim S.-W., Ju Y.H., Han S., Kim J.S., Lee H.-J., Han C.J., Lee C.-R., Jung S.-B., Kim Y., Kim J.-W. (2019). A UV-responsive pressure sensitive adhesive for damage-free fabrication of an ultrathin imperceptible mechanical sensor with ultrahigh optical transparency. J. Mater. Chem. A.

[16] Ebe K., Seno H., Horigome K. (2003). UV curable pressure-sensitive adhesives for fabricating semiconductors. I. Development of easily peelable dicing tapes. J. Appl. Polym. Sci..

[17] Horigome K., Ebe K., Kuroda S. (2004). UV curable pressure-sensitive adhesives for fabricating semiconductors. II. The effect of functionality of acrylate monomers on the adhesive properties. J. Appl. Polym. Sci..

[18] Zosel A. (1998). The effect of fibrilation on the tack of pressure sensitive adhesives. Int. J. Adhes. Adhes..

[19] Horgnies M., Darque-Ceretti E., Felder E. (2007). Relationship between the fracture energy and the mechanical behaviour of pressure-sensitive adhesives. Int. J. Adhes. Adhes..

[20] Yamamura K., Fujii S., Nakamura Y., Fujiwara K., Hikasa S., Urahama Y. (2013). Influence of diblock addition on tack in a polyacrylic triblock copolymer/tackifier system measured using a probe tack test. J. Appl. Polym. Sci..

[21] Nakamura Y., Sakai Y., Imamura K., Ito K., Fujii S., Urahama Y. (2012). Effects of the compatibility of a polyacrylic block copolymer/tackifier blend on the phase structure and tack of a pressure-sensitive adhesive. J. Appl. Polym. Sci..

[22] Achilias D.S., Karabela M.M., Varkopoulou E.A., Sideridou I.D. (2012). Cure Kinetics Study of Two Epoxy Systems with Fourier Tranform Infrared Spectroscopy (FTIR) and Differential Scanning Calorimetry (DSC). J. Macromol. Sci. A.

[23] Lu X., Cao G., Niu Z., Pan Q. (2014). Viscoelastic and adhesive properties of single-component thermo-resistant acrylic pressure sensitive adhesives. J. Appl. Polym. Sci..

[24] Gdalin B.E., Bermesheva E.V., Shandryuk G.A., Feldstein M.M. (2011). Effect of Temperature on Probe Tack Adhesion: Extension of the Dahlquist Criterion of Tack. J. Adhesion.

[25] Lee S.-W., Park J.-W., Kim H.-J., Kim K.-M., Kim H.-I., Ryu J.-M. (2012). Adhesion Performance and Microscope Morphology of UV-Curable Semi-interpenetrated Dicing Acrylic PSAs in Si-Wafer Manufacture Process for MCP. J. Adhes. Sci. Technol..

[26] Ozawa T., Ishiwata S., Kano Y., Kasemura T. (2000). Acrylate Copolymer/Ultraviolet Curable Oligomer Blends as Pressure-sensitive Adhesives. J. Adhesion.

[27] Sinclair R.C., Suter J.L., Coveney P.V. (2019). Micromechanical exfoliation of graphene on the atomistic scale. PCCP.

[28] Taherian F., Marcon V., van der Vegt N.F.A., Leroy F. (2013). What Is the Contact Angle of Water on Graphene?. Langmuir.

[29] de Torre L.E.C., Bottani E.J., Martínez-Alonso A., Cuesta A., García A.B., Tascón J.M.D. (1998). Effects of oxygen plasma treatment on the surface of graphitized carbon black. Carbon.

[30] Cai A., Yang L.-p., Chen J.-p., Xi T.-g., Xin S.-g., Wu W. (2010). Thermal Conductivity of Anodic Alumina Film at (220 to 480) K by Laser Flash Technique. J. Chem. Eng. Data.

[31] Gresil M., Wang Z., Poutrel Q.-A., Soutis C. (2017). Thermal Diffusivity Mapping of Graphene Based Polymer Nanocomposites. Sci. Rep..

[32] Sadej M., Gojzewski H., Gajewski P., Vancso G.J., Andrzejewska E. (2018). Photocurable acrylate-based composites with enhanced thermal conductivity containing boron and silicon nitrides. Express Polym. Lett..

