# Supplementary material for: Thermally Conductive Film Fabricated Using Perforated Graphite Sheet and UV-Curable Pressure-Sensitive Adhesive
Source: Nanomaterials (Basel). 2021 Jan 3;11(1):93. doi: 10.3390/nano11010093 (PMC7824192; doi:10.3390/nano11010093)
Supplement: Supplementary file 1 [file nanomaterials-11-00093-s001.pdf]

nanomaterials

MDPI

Supplementary Materials

Thermally Conductive Film Fabricated Using Perforated Graphite Sheet and UV-Curable Pressure-Sensitive Adhesive

Hee-Jin Lee, Gayoung Lim, Eunseong Yang, Young-Seok Kim, Min-Gi Kwak and Youngmin Kim *

Display Research Center, Korea Electronics Technology Institute, 25 Saenariro, Bundang-gu, Seongnam 13509, Korea; gmlwls5010@gmail.com (H.-J.L.); addzero@kakao.com (G.L.); didguswjd567@naver.com (E.Y.); vis4freedom@keti.re.kr (Y.-S.K.); kwakmg@keti.re.kr (M.-G.K.)

* Correspondence: ymkim@keti.re.kr

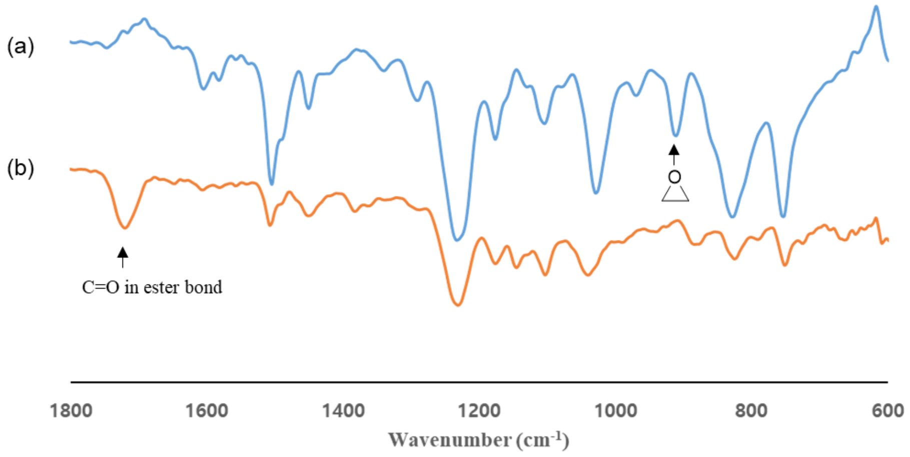

Figure S1. FTIR spectra of (**a**) tris(4-hydroxyphenyl)methane triglycidyl ether and (**b**) AAE−2.

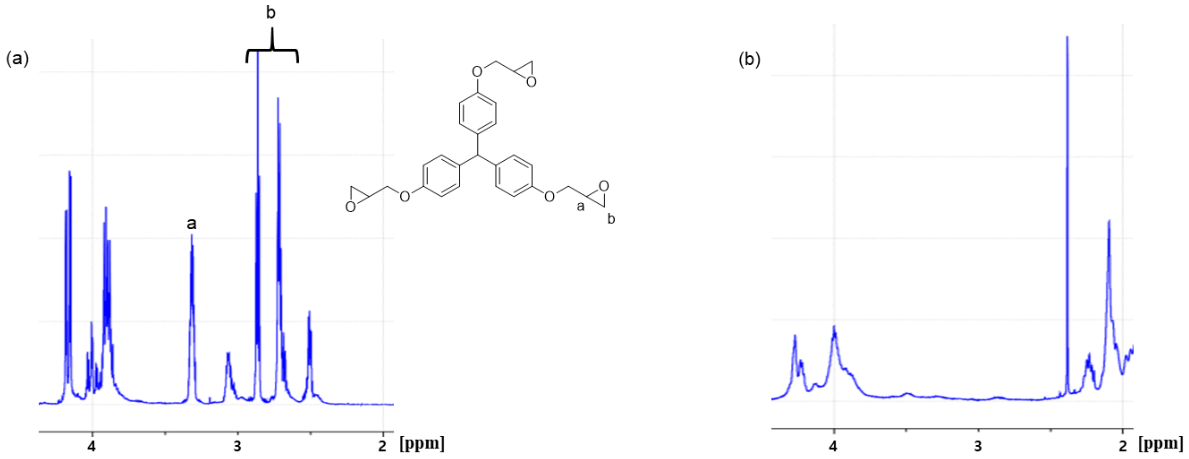

Figure S2. ^{1}H-NMR spectra of (**a**) tris(4-hydroxyphenyl)methane triglycidyl ether and (**b**) AAE−2. The proton resonances for epoxide ring were disappeared through a ring-opening reaction.

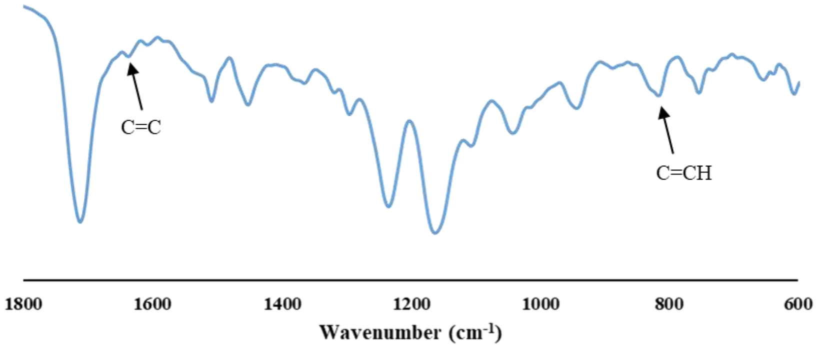

Figure S3. FTIR spectra of TM−3.

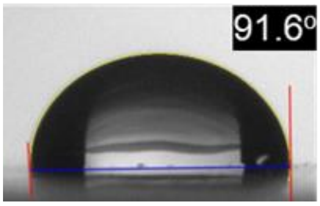

Figure S4. A photography image of contact angle of a water drop on the graphite sheet.

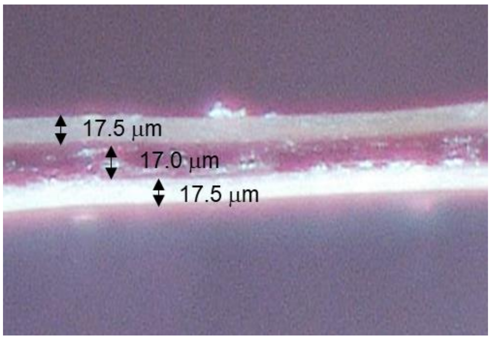

Figure S5. A cross sectional microscopic image of the g−TC film.

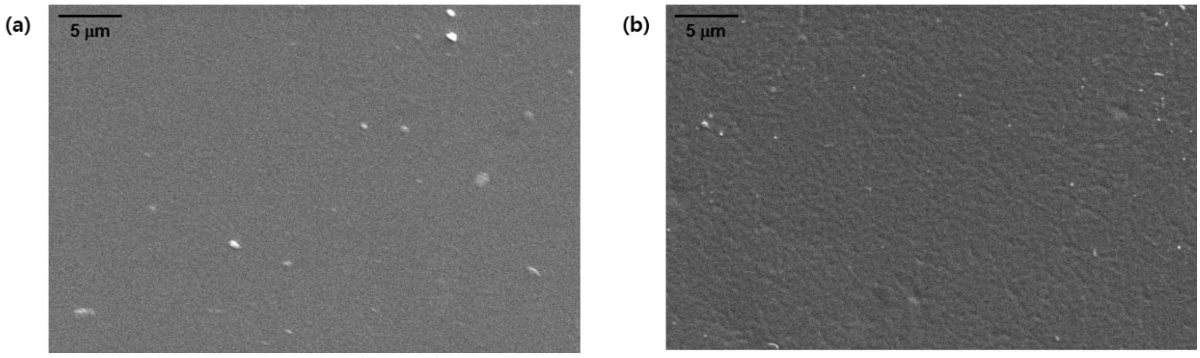

Figure S6. SEM images of surfaces of the g−TC films (**a**) before and (**b**) after U-folding tests.
